# Genome-Wide Methylation Analysis Identifies Specific Epigenetic Marks In Severely Obese Children

**DOI:** 10.1038/srep46311

**Published:** 2017-04-07

**Authors:** Delphine Fradin, Pierre-Yves Boëlle, Marie-Pierre Belot, Fanny Lachaux, Jorg Tost, Céline Besse, Jean-François Deleuze, Gianpaolo De Filippo, Pierre Bougnères

**Affiliations:** 1 INSERM U1169, Bicêtre Hospital, Paris Sud University, Le Kremlin-Bicêtre, France; 2 Pierre et Marie Curie University, INSERM, Paris, U707, France; 3 Laboratory for Epigenetics and Environment, Centre National de Génotypage, CEA - Institut de Génomique, Evry, France; 4 Centre National de Génotypage, CEA - Institut de Génomique, Evry, France; 5 Department of Pediatric Endocrinology, Bicêtre Hospital, Paris Sud University, Le Kremlin-Bicêtre, France

## Abstract

Obesity is a heterogeneous disease with many different subtypes. Epigenetics could contribute to these differences. The aim of this study was to investigate genome-wide DNA methylation searching for methylation marks associated with obesity in children and adolescents. We studied DNA methylation profiles in whole blood cells from 40 obese children and controls using Illumina Infinium HumanMethylation450 BeadChips. After correction for cell heterogeneity and multiple tests, we found that compared to lean controls, 31 CpGs are differentially methylated in obese patients. A greatest proportion of these CpGs is hypermethylated in obesity and located in CpG shores regions. We next focused on severely obese children and identified 151 differentially methylated CpGs among which 10 with a difference in methylation greater than 10%. The top pathways enriched among the identified CpGs included the “IRS1 target genes” and several pathways in cancer diseases. This study represents the first effort to search for differences in methylation in obesity and severe obesity, which may help understanding these different forms of obesity and their complications.

Data from U.S. population surveys demonstrate a significant increase in obesity prevalence among children age 2–19 years old, from 5.5% in 1976–1980^1^ to 16.9% in 2007–2010^1^,^2^, with obesity defined as body mass index (BMI) ≥ 95th percentile using the Centers for Disease Control and Prevention (CDC)^3^. Severe obesity is the most rapidly growing paediatric obesity subgroup, and recent estimates suggest that this disease afflicts up to 6% of all children and adolescents in the United States^4^. Compared to youth with BMI in the obese range, those with severe obesity have higher rates of immediate and long-term metabolic and cardiovascular comorbidities^5^. It stands to reason that youth with obesity and severe obesity may also differ in aetiological factors and consequences, including epigenetic.

There is growing evidence that DNA methylation might contribute to obesity. Indeed, candidate gene methylation studies in animal models and humans have demonstrated methylation changes in promoters of various genes that are implicated in obesity, appetite control and/or metabolism, insulin signaling, immunity, growth and circadian clock regulation^6^,^7^,^8^,^9^. For example, the methylation percentage of *insulin-like growth factor 2 (IGF2*) promoter was higher in overweight infants than in lean infants^8^. The methylation of *peroxisomal proliferator activated receptor-γ-co-activator-1α* promoter in children blood predicts adiposity at adolescence independently of sex, age, pubertal timing and activity^6^.

To identify novel genes and pathways related to obesity and obesity-induced complications, epigenome-wide association studies (EWAS) are needed. Two previous studies using the HumanMethylation27 BeadChip with 27,000 CpGs, primarily targeting gene promoters and CpG islands (CGIs), examined blood leukocytes of obese and lean adolescents^10^,^11^. Wang *et al*. discovered two obesity-associated inflammatory genes *Ubiquitin Associated And SH3 Domain Containing A* and *Tripartite Motif Containing 3 (UBASH3A* and *TRIM3*), and Almen *et al*. identified 20 CpGs differentially methylated between groups. A larger study looking at the impact of BMI on DNA methylation in different tissues using the HumanMethylation 450 BeadChip by Dick *et al*. found five probes correlated with BMI, three of which were in the intron of *HIF3A (Hypoxia Inducible Factor 3, Alpha Subunit*)^12^. In children, two recent papers also showed that specific DNA methylation profiles in blood differ between lean and obese subjects^13^,^14^. Using the NimbleGen Human DNA Methylation 385 K Promoter Plus CpG Island Microarray, Ding *et al*. found 575 demethylated CGIs and 277 hypermethylated CGIs in obese children. Finally, Huang *et al*. identified 129 differentially methylated CpGs associated with 80 unique genes. None of the obesity-associated CpGs was common to all studies, which may be due to differences between the study populations, diverse genetic backgrounds, or heterogeneous metabolic phenotypes between different BMI categories.

Given the paucity of research on the different BMI categories, the purpose of this study were twofold: (i) investigate DNA methylation marks in all obese children compare to lean controls; (ii) identify the differentially methylated CpGs associated with severe obesity in childhood. We hypothesized that patients with severe obesity could help at identifying epigenetic changes, as extreme phenotypes improve genetic association studies.

## Results

### Identification of differentially methylated CpGs associated with childhood obesity

The genome-wide methylation analysis was conducted in 20 obese children (BMI Z-score > 2.5,) and 17 controls (Table 1). Three controls were excluded due to bad quality DNA and arrays.

Because DNA methylation varies by cell type and could bias EWAS results conducted in blood samples, we estimated the cell type compositions in each sample using minfi, and found that cellular composition was not similar between cases and control subjects (Supplemental Table 1). To correct for these differences, we used next the Houseman’s correction algorithm^15^.

**Figure 1:**
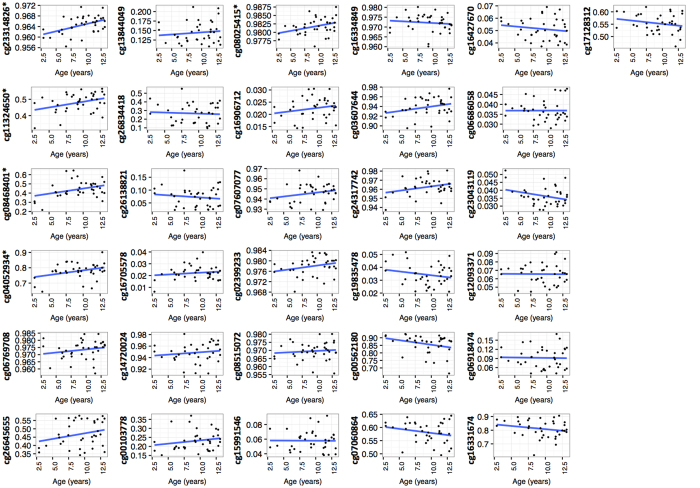
Correlation between DNA methylation and age in 20 obese children and 17 controls at the 31 associated CpGs. *Significant correlation (p < 0.05), DNA methylation value in the y-axis and age in years in the x-axis.

**Figure 2:**
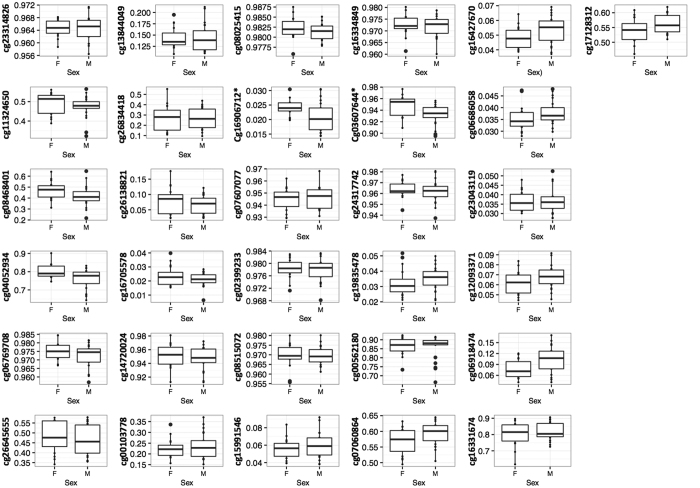
Association between DNA methylation and sex in 20 obese children and 17 controls at the 31 associated CpGs. *Significant correlation (p < 0.05), DNA methylation value in the y-axis and sex (M = male, F = female) in the x-axis.

After correction for cellular heterogeneity and multiple testing, thirty-one CpGs were differentially methylated (FDR ≤ 0.05) between obese children and control subjects, 10 were hypermethylated, and 21 were hypomethylated in obese compared to lean children (Table 2). The largest difference was observed for cg26834418 located in the promoter region (TSS1500) of the *CHORDC1* gene (+13% in control subjects compare to obese children). This gene also showed two other probes differentially methylated in obese compare to lean children. Because of the relatively small numbers in the study and small differences in methylation levels for most of the significant CpG sites, we analysed the confounding variables age and sex. We found that the methylation of 5/31 and 1/31 of the identified CpG sites was correlated with respectively age or sex (Figs 1 and 2).

**Figure 3:**
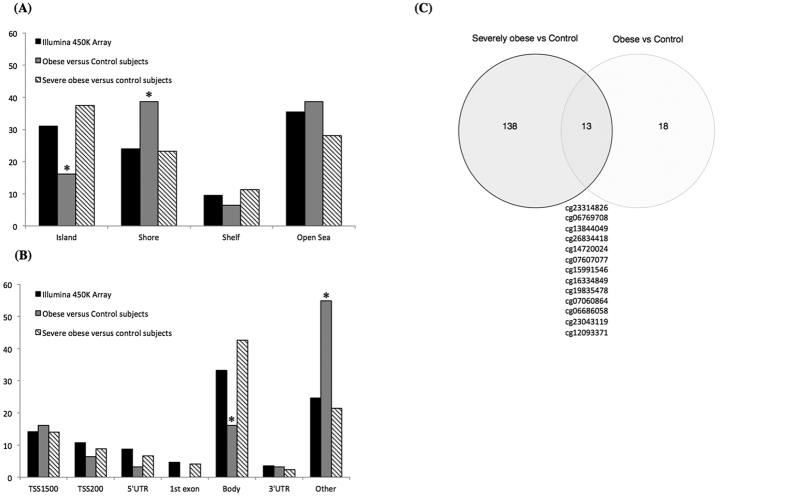
Distribution of differentially methylated CpGs versus all CpGs sites on the Infinium HumanMethylation450 BeadChip in relation to (**A**) CpG island regions; (**B**) the nearest gene regions. Chi-square analysis was performed to test over- or under-representation of sequence features among the differentially methylated CpGs, *P.value < 0.05. (**C**) Venn diagram of the identified CpGs in obese or severely obese children, below the 13 common CpGs.

The distribution of the 31 CpGs showed that DNA methylation variation was distributed over the CpG island shores, and that although CGI are enriched on the array (31% of Illumina probes are in CGI); only 16% of our obesity–associated CpGs were located in CGI, compared to 38% in shores (22% in S-Shore and 16% in N-shore) (p = 0.016, Pearson’s Chi-squared test) (Fig. 3A). The genomic distribution of the 31 CpGs in comparison to all the probes located on the 450 K BeadChip array with respect to gene structure is shown in Fig. 3B. We also found an enrichment of differentially methylated CpGs outside promoter and gene body (p = 2.10^−4^, Pearson’s Chi-squared test) (Fig. 3B).

Since obesity is an extremely heterogeneous disease, we then chose to focus only on the extremely obese children (BMI z-score ≥ 3.5).

### Identification of differentially methylated CpGs associated with severe childhood obesity

We analysed next DNA methylation marks only in the 11 severely obese children of the group. We found 151 differentially methylated CpGs (q.value ≤ 0.05), 69 were hypermethylated, and 82 were hypomethylated in severely obese patients compared to lean controls (Supplemental Table 2). Of these 151 differentially methylated CpGs, ten had a greater than 10% difference in methylation between the case and control groups. The most significant difference was observed for a cg27590049 located in the *LMX1A (LIM Homeobox Transcription Factor 1, Alpha)*^16^; and the largest difference was observed for a cg07944420 located on the gene body of *ACSF3 (Acyl-CoA Synthetase Family Member 3)* that showed a decreased by 17% of methylation level in control subjects compared to obese children. Thirteen of the 151 CpGs were common to our first analysis comparing obese children all together, 18 seems specific to moderately obese children and 138 to severely obese children (Fig. 3C).

Next, we performed a gene set enrichment analysis (GSEA) to explore the potential of shared biologically relevant pathways among the obesity-associated methylation events. Seven pathways showed a significant enrichment including “IRS1 Target Genes” and different cancer traits (Table 3).

The genomic distribution of these 151 differentially methylated CpGs in relation to CpG density (CGIs, shores, shelves, and open sea) was not clearly different from the whole array CpG distribution and there was no significant enrichment within specific gene regions (data not shown).

## Discussion

In this study we aimed to identify obesity related methylation marks in peripheral blood leukocytes using a genome wide approach in youth obese children. The primary finding of this study was that most of the epigenetic marks are different in moderate and severe obesity. We identified respectively 18 and 138 differentially methylated CpGs between moderate or severe obese children and lean controls. As observed for genetic association studies, sampling individuals with extreme phenotypes can enrich the presence of epigenetic variations and can therefore lead to an increase in detection of these differences. Moreover, most of the differentially methylated CpGs was found within open seas or intergenic regions, consistently with previous findings showing that DNA methylation may be more dynamic outside CGIs.

Compare to previous studies^10^,^11^,^12^,^13^,^17^,^18^, we replicated the association between DNA methylation level and obesity at 10 gene loci (*ANKRD11: ankyrin repeat domain 11, AVPI1: arginine vasopressin-induced 1, CDK19: cyclin-dependent kinase 19, FOXK2: forkhead box K2, HDAC4: histone deacetylase 4, IFT140: intraflagellar transport 140, KIAA0753, LTBP1: latent transforming growth factor beta binding protein 1, MYOM2: myomesin 2, TBC1D8: TBC1 domain family, member 8*). Among these genes, *HDAC4* is an interesting candidate since recently, Abu-Farha *et al*. showed a reduced expression of *HDAC* mRNA and protein in human obese subjects both in blood cells and adipose tissue^19^. This is consistent with clinical data in humans that associated the haploinsufficiency of HDAC4 with obesity^20^. In our study, we found a *HDAC* methylation level higher in obese children than in controls.

Numerous genes found in our severe obese analysis were also associated with cancer. Many epidemiological and clinical studies have demonstrated that early obesity is an established risk factor for many cancers in later life^21^. Cross talk between macrophages, adipocytes, and epithelial cells occurs *via* obesity-associated hormones, growth factor signalling, inflammation, vascular integrity processes, microenvironmental perturbations, and other mediators, which could enhance the cancer risk and/or progression^22^. Thus, the methylation changes in childhood obesity could increase the risks for later cancer susceptibility.

Most of the identified genes are not expressed or do not have a relevant function in blood cells; whether these epigenetic marks in blood may reflect or correlate with methylation in more relevant tissues is not known. However, several studies showed that DNA methylation measured in whole blood is a marker for less accessible tissues that are directly involved in disease. For instance, Murphy *et al*. have shown for example that methylation across the H19 DMRs (Differentially Methylated Regions) was similar across several tissues from divers embryonic origins^23^. Likewise, in non-imprinted loci, Talens *et al*. also found that DNA methylation levels did not differ in blood and buccal cells, from mesodermal and ectodermal embryonic tissues, respectively^24^. The recent work of Huang *et al*.^25^ identified 1,285 discordant and 1,961 concordant genes for methylation between blood and adipose tissue; the discordant genes are enriched in biological functions related to immune response, leukocyte activation or differentiation, and blood coagulation. Moreover, epigenetic marks associated with type-2 diabetes^26^ or adiposity^7^ have also been identified in peripheral tissues.

There were several limitations to this study. The first limitation was that we used DNA from whole blood. To correct our methylation data for this weakness, we used a Houseman correction algorithm^27^. It must also be noted that adjusting for cell composition makes impossible the process of replication and validation of the identified CpGs by pyrosequencing. Replication could also be accomplished if there exists an independent replication population; in this case the models could be re-applied. We tried to replicate our findings by running the available datasets for common obesity (GEO DataSets: GSE25301, GSE43975, GSE44763, GSE73103) under the RefFreeEwas procedure, but we failed to find any associated CpGs after correction for cell heterogeneity, even those previously identified by the authors without cell heterogeneity correction.

The second limitation is that we cannot conclude about the existence of these epigenetic marks before the establishment of the obesity. While this lack of interpretability is inherent to the design of the cross-sectional case control study, the finding of methylation marks associated with the early stages of severe obesity in young patients may be of pathogenic relevance to certain features or complications of this disease. Indeed, the marks that have been found here could be used in a longitudinal study of the young patients in order to gain both biomarker and mechanistic insights. The longitudinal sampling of cells from adolescence to adulthood should further allow which of these epigenetic changes follow the development of the long term overt phenotype of severe obesity and its complications.

Our major strength was that all studied participants were children, aged from 3 to 13 years old, less subject to cofounding factors like medication or comorbidities, very common in adult obese patients.

In conclusion, the identification of methylation changes in specific genes will provide important targets for further study into the underlying mechanisms and the therapeutic potential for childhood obesity.

## Methods

### Study participants

Twenty obese children (5 to 13 years old) and equal numbers of control children (3 to 13 years old) were included in the study. The BMI for obese children (10 male) and lean children (11 male) was 26.0 ± 4.8 kg/m^2^ and 16.7 ± 2.2 kg/m^2^, respectively (Table 1). Age and gender-specific BMI Z-scores were determined by using the growth charts form the World Health Organization with a mean BMI Z-score of 3.6 ± 0.8 for obese children and 0.2 ± 1.1 for controls. Patients with monogenic or syndromic forms of obesity were excluded. To limit the risk of population stratification, all recruited children are of Caucasian ancestry assessed by family history and grandparents’ birthplace. All methods were carried out in accordance with relevant guidelines and regulations. Patients and controls were included in the study according to the French bioethics law with families being carefully informed and having signed a detailed informed consent. All protocols were agreed by French ethic boards (CODECOH DC-2013-1977, CPP C0-13-004, CCTIRS n°14-116bis, CNIL n°91 4228).

### Infinium humanMethylation450 beadchip array

DNA was extracted from whole blood cells of 20 case and 20 control subjects using Gentra DNA extraction kit (Qiagen). Genomic DNA (1 ug) from each of the 40 subjects was bisulphite-converted using Zymo EZ DNA Methylation-Gold kit (ZymoResearch) and the DNA was analysed using the Infinium HumanMethylation450 platform (Illumina, Inc.) by The Genotyping National Center (CNG, CEA, Evry, France).

### Infinium HumanMethylation450 BeadChip array data processing

DNA methylation status of case and control subjects was established using Illumina Infinium HumanMethylation450 BeadChips that cover 485,764 cytosine positions of the human genome. Preprocessing and normalization involved steps of probe filtering, color bias correction, background subtraction and subset quantile normalization as previously described^28^. After these intra-sample normalization procedures, β-values were calculated. To avoid batch effect, all samples were processed together. Obese subjects were compared to control subjects, using t-tests.

### Assessment of cell composition differences

The R package minfi^29^ was used to estimate the fraction of CD8T-, CD4T-, NK- and B-cells, monocytes, and granulocytes in our 40 samples. This package allows for estimating cell fractions in Illumina 450 K methylation data from whole blood, based on the methylation data published for flow-sorted cells^30^, and algorithms^27^.

### Cell heterogeneity correction for the methylation data analysis

To correct our methylation data analysis for cell heterogeneity between samples, we used R package RefFreeEwas^15^. This package allows for conducting EWAS while deconvoluting DNA methylation arising as mixtures of cell types. This method is similar to surrogate variable analysis, except that it makes additional use of a biological mixture assumption.

## Additional Information

**How to cite this article:** Fradin, D. *et al*. Genome-Wide Methylation Analysis Identifies Specific Epigenetic Marks In Severely Obese Children. *Sci. Rep.* **7**, 46311; doi: 10.1038/srep46311 (2017).

**Publisher's note:** Springer Nature remains neutral with regard to jurisdictional claims in published maps and institutional affiliations.

## Supplementary Material

Supplementary Information

Supplementary Dataset

## Figures and Tables

**Table 1:** Characteristic of obese and control children.

ID	Sex	Age (years)	Weight (kg)	Height (m)	BMI (kg/m2)	BMI Z-score	Birth Weight (g)	Birth Height (cm)	Term (weeks)	Physical activity*	Physical activity (hr/wk)*	Dietary intake (kcal/day)**
**ob1**	M	12.02	64.9	1.54	27.36	3.58	4400	54	41	YES	2.5	3230
**ob2**	M	11.48	74	1.59	29.27	4.15	3570	52.5	40	YES	4	2800
**ob3**	M	13.12	89	1.65	32.69	4.31	4310	52.5	39	YES	5	3000
**ob4**	F	7.62	32	1.3	18.93	2.9	2450	44	34	YES	1	1910
**ob5**	F	12.82	81.6	1.56	33.53	4.32	2900	48	40	YES	1.5	—
**ob6**	F	13.11	66.6	1.61	25.69	2.58	3300	51	41	YES	6.5	—
**ob7**	M	12.79	87.9	1.56	36.12	4.96	3700	50	40	YES	4	2900
**ob8**	F	12.06	60	1.57	24.34	2.6	2790	46.5	36	YES	1	2815
**ob9**	F	11.55	60.9	1.49	27.43	3.59	3610	51.5	41	NO	—	3130
**ob10**	F	9.53	46.6	1.37	24.83	3.85	3250	50	41	YES	1	1660
**ob11**	M	5.49	28	1.21	19.12	2.71	4315	52.5	41	YES	1	1800
**ob12**	M	12.48	69	1.45	32.82	4.48	3100	—	36	YES	2	—
**ob13**	F	8.34	36	1.29	21.63	3.4	4230	52.5	41	YES	1	2080
**ob14**	M	8.58	58.5	1.53	24.99	4.34	3050	48	40	YES	6	—
**ob15**	M	9.46	53.6	1.41	26.96	4.43	3950	51	40	YES	6.5	2200
**ob16**	M	9.17	42	1.34	23.39	3.56	3300	49	—	YES	2	2400
**ob17**	F	7.53	35.5	1.3	21.01	3.32	3310	50.5	39	YES	7	2550
**ob18**	F	9.72	37.7	1.33	21.31	2.64	3430	49	39	YES	2	1954
**ob19**	M	11.77	58.3	1.49	26.26	3.49	2760	46	36	NO	—	2400
**ob20**	F	10.42	46.3	1.43	22.64	2.91	2660	47	38	YES	5	2200
**Ctrl1**	M	6.25	21	1.2	14.58	−0.74	2790	49	—	—	—	—
**Ctrl2**	F	9.28	47	1.55	19.56	2.09	3650	52.5	—	—	—	—
**Ctrl3**	F	11.23	52	1.62	19.81	1.46	2960	47	—	—	—	—
**Ctrl4**	M	11.52	38	1.4	19.39	1.23	2800	—	—	—	—	—
**Ctrl5**	F	13.00	41	1.51	17.98	−0.07	2700	47	—	—	—	—
**Ctrl6**	F	12.84	48	1.58	19.23	0.67	—	—	—	—	—	—
**Ctrl7**	M	7.45	23.5	1.23	15.53	0	—	—	—	—	—	—
**Ctrl8**	F	6.03	22	1.22	14.78	−0.31	3560	49.5	—	—	—	—
**Ctrl9**	M	6.66	25.1	1.2	17.43	1.45	3550	50	—	—	—	—
**Ctrl10**	M	10.00	37	1.49	16.66	0.19	2900	—	—	—	—	—
**Ctrl11**	F	11.15	37	1.45	17.59	0.45	—	—	—	—	—	—
**Ctrl12**	M	7.59	28.2	1.32	16.18	0.41	—	—	—	—	—	—
**Ctrl13**	F	5.95	15	1.08	12.86	−2.08	3040	49	—	—	—	—
**Ctrl14**	M	9.27	30	1.4	15.31	−0.51	2630	52	—	—	—	—
**Ctrl15**	M	10.16	32	1.4	16.33	−0.02	3200	52	—	—	—	—
**Ctrl16**	M	11.28	33	1.39	17.08	0.2	—	—	—	—	—	—
**Ctrl17**	M	2.90	12	0.92	14.18	−1.91	3260	51	—	—	—	—

*Data obtained from a self-completed questionnaire; **A 24-hour dietary recall was conducted for all patients by a dietitian, based on individual interview with parents or caregivers.

**Table 2:** Differentially methylated CpGs in obese children.

Probes	CHR	UCSC RefGene Name	UCSC RefGene Group	Relation to UCSC CpG Island	Methylation percent in obese children	Methylation percent in control subjects	p.value	q.value
cg23314826	8				0.962 ± 0.003	0.965 ± 0.003	1.31e-08	0.003
cg11324650	3			Island	0.449 0.056	0.521 0.034	4.59e-08	0.005
cg08468401	3				0.390 0.070	0.501 0.087	7.09e-08	0.005
cg04052934	6				0.752 0.050	0.796 0.052	2.29e-07	0.012
cg06769708	20	*DLGAP4*	Body	S_Shore	0.970 0.007	0.976 0.005	2.05e-07	0.012
cg19835478	21	*COL6A1*	TSS1500	Island	0.039 0.007	0.029 0.006	2.78e-07	0.013
cg26645655	3				0.426 0.062	0.502 0.071	3.23e-07	0.013
cg00562180	16			N_Shelf	0.891 0.021	0.793 0.110	5.87e-07	0.018
cg07060864	16				0.604 0.024	0.558 0.038	6.01e-07	0.018
cg13844049	1				0.131 0.018	0.166 0.045	6.36e-07	0.018
cg26834418	11	*CHORDC1*	TSS1500	S_Shore	0.206 0.087	0.324 0.095	7.21e-07	0.019
cg16427670	13	*ARHGEF7*	TSS1500		0.056 0.007	0.046 0.007	9.24e-07	0.021
cg16705578	3			Island	0.019 0.004	0.025 0.005	9.19e-07	0.021
cg26138821	11	*CHORDC1*	TSS200	S_Shore	0.054 0.031	0.093 0.031	9.61e-07	0.021
cg14720024	5			S_Shore	0.939 0.015	0.961 0.010	1.09e-06	0.023
cg00103778	20			N_Shore	0.202 0.028	0.264 0.054	1.36e-06	0.027
cg06686058	11	*ADM*	5′UTR;1stExon	N_Shore	0.039 0.005	0.034 0.004	1.44e-06	0.027
cg08025415	19	*TLE2*	Body	N_Shelf	0.981 0.002	0.982 0.002	1.48e-06	0.027
cg16906712	6	*HIST1H2BG;HIST1H2AE*	TSS1500;3′UTR;1stExon	S_Shore	0.019 0.004	0.024 0.003	1.53e-06	0.027
cg23043119	5	*STARD4*	TSS1500	S_Shore	0.039 0.006	0.033 0.005	1.61e-06	0.027
cg07607077	7	*C7orf20*	Body	S_Shore	0.941 0.009	0.950 0.005	1.71e-06	0.027
cg02399233	11	*NADSYN1*	Body		0.977 0.004	0.979 0.003	2.23e-06	0.032
cg08515072	8			N_Shore	0.967 0.005	0.971 0.005	2.16e-06	0.032
cg12093371	10			N_Shore	0.073 0.009	0.058 0.010	2.16e-06	0.032
cg06918474	5	*MARCH3*	3′UTR		0.110 0.031	0.084 0.049	2.38e-06	0.034
cg16331674	22			Island	0.843 0.048	0.785 0.063	2.56e-06	0.035
cg15991546	1	*DPYD*	TSS200	Island	0.050 0.009	0.068 0.013	2.82e-06	0.036
cg17128312	19	*FBXW9*	Body	N_Shore	0.572 0.028	0.537 0.036	2.79e-06	0.036
cg16334849	8				0.970 0.004	0.973 0.005	3.19e-06	0.040
cg03607644	8				0.929 0.018	0.950 0.014	3.88e-06	0.045
cg24317742	1				0.958 0.007	0.967 0.008	4.32e-06	0.048

Table listing the differentially methylated CpG probes with corresponding UCSC RefGene Name, group and group related to CpG Island, methylation percent in obese and control children, p.values and q.values after cell type estimate adjustments.

**Table 3:** Gene set enrichment analysis of severe obese children associated CpGs.

Gene Set Name	Genes in Gene Set	Description of the Set	Genes in Overlap (n)	Genes in Overlap (Name)	p.value	FDR q.value
TUMOR ZONE PERIPHERA VS CENTRAL DN	634	Down-regulated genes in peripheral zone of human pancreatic cancer growing in the pancreas of nude mice compared to that of the tumor from the central zone	8	*GMDS FAM59A COL6A1 P4HB NXPH4 RAB3D FAT2 KIAA1530*	6.22e-6	1.78e-2
ENDOCRINE THERAPY RESISTANCE 3	720	The ‘group 3 set’ of genes associated with acquired endocrine therapy resistance in breast tumors expressing ESR1 and ERBB2	8	*GMDS FAM59A FBN2 SLC7A14 TMEM45B ATP2C2 ACVR2A STARD4*	1.56e-5	1.78e-2
ADULT TISSUE STEM MODULE	721	The ‘adult tissue stem’ module: genes coordinately up-regulated in a compendium of adult tissue stem cells	8	*COL6A1 ID4 COL1A1 HLX FLOT1 DIP2C CBFA2T3 EPHB4*	1.57e-5	1.78e-2
IRS1 TARGETS UP	113	Up-regulated in brown preadipocytes with IRS1 knockout vs wild type controls; the knockouts have severe defects in adipocyte differentiation	4	*FBN2 CYBA WNT10A HSPA4*	2.97e-5	2.34e-2
LIVER CANCER SUBCLASS S1	237	Genes from ‘subtype S1′ signature of hepatocellular carcinoma (HCC): aberrant activation of the WNT signaling pathway	5	*COL6A1 CYBA ITPR3 TAX1BP3 RPA2*	3.45e-5	2.34e-2
COLON CANCER UP	871	Up-regulated genes in colon carcinoma tumors compared to the matched normal mucosa samples	8	*GMDS CYBA ITPR3 COX6B1 THOP1 SH3GL1 DLGAP4 SHFM1*	5.98e-5	3.39e-2

This table displays gene pathways overrepresented among the 151 differentially methylated CpGs (FDR q.value < 0.05).
