# Supplementary material for: Genome-Wide Methylation Analysis Identifies Specific Epigenetic Marks In Severely Obese Children
Source: Sci Rep. 2017 Apr 7;7:46311. doi: 10.1038/srep46311 (PMC5384222; doi:10.1038/srep46311)
Supplement: Supplementary Information [file srep46311-s1.pdf]

GENOME-WIDE METHYLATION ANALYSIS IDENTIFIES SPECIFIC EPIGENETIC MARKS IN SEVERELY OBESE CHILDREN.

Delphine Fradin, Pierre-Yves Boëlle, Marie-Pierre Belot, Fanny Lachaux, Jorg Tost, Céline Besse, Jean-François Deleuze, Gianpaolo De Filippo and Pierre Bougnères

Supplemental Table 1. Comparison of cell proportion estimates according the obesity status.

	Est	StdErr0	StdErr1	StdErr2	Zscore	Pvalue
<Intercept>	0.01681	0.2104	0.09922	0.1668	0.1008	9.197e-01
CD8T	11.41147	1.0834	2.38473	2.4220	4.7115	2.459e-06
CD4T	4.56394	1.1948	3.49980	3.6333	1.2561	2.091e-01
NK	4.15629	0.6964	1.89673	2.0380	2.0394	4.141e-02
Bcell	4.99052	0.8589	2.63872	2.6585	1.8772	6.049e-02
Mono	-4.91431	0.8241	3.32222	3.3143	-1.4828	1.381e-01
Gran	-20.56218	2.4226	7.75357	8.5588	-2.4025	1.628e-02
